# A Fully Integrated Paper-Microfluidic Electrochemical Device for Simultaneous Analysis of Physiologic Blood Ions

**DOI:** 10.3390/s18010104

**Published:** 2018-01-01

**Authors:** Joon-Hyung Jin, Joon Hyub Kim, Sang Ki Lee, Sam Jin Choi, Chan Won Park, Nam Ki Min

**Affiliations:** 1 Department of Chemical Engineering, Kyonggi University, 154-42 Gwanggyosan-ro, Yeongtong-gu, Suwon-si, Gyeonggi-do 16227, Korea; jjh1023@kgu.ac.kr; 2 Department of Electro-Mechanical Systems Engineering, Korea University, 2511 Sejong-ro, Sejong City 339-770, Korea; kim4539@korea.ac.kr (J.H.K.); snagki0@korea.ac.kr (S.K.L.); 3 Department of Biomedical Engineering, College of Medicine, Kyung Hee University, Seoul 130-701, Korea; medchoi@khu.ac.kr; 4 Department of Electrical and Electronic Engineering, Kangwon National University, Chuncheon 200-701, Korea

**Keywords:** blood ion analysis, cation permeable film, electrochemical sensor, integrated paper microfluidic device, potentiometric sensor

## Abstract

A fully integrated paper microfluidic electrochemical device equipped with three different cation permeable films is developed to determine blood ions (Cl^−^, Na^+^, K^+^, and Ca^2+^) at a time. These blood ions that are normally dissolved in the real human blood stream are essential for cell metabolisms and homeostasis in the human body. Abnormal concentration of blood ions causes many serious disorders. The optimized microfluidic device working without any external power source can directly and effectively separate human blood components, and subsequently detect a specific blood ion with minimized interference. The measured sensitivity to Cl^−^, K^+^, Na^+^, and Ca^2+^ are −47.71, 45.97, 51.06, and 19.46 in mV decade^−1^, respectively. Potentiometric responses of the microfluidic devices to blood serum samples are in the normal ranges of each cation, and comparable with responses from the commercial blood ion analyzer Abbott i-Stat.

## 1. Introduction

A portable medical system with hospital quality accuracy is highly desirable for the rapid diagnosis of infectious lethal diseases in the field. Such highly contagious viral diseases as the Middle East respiratory syndrome, severe acute respiratory syndrome, and Ebola commonly show that they spread rapidly by person-to-person contact, mostly in public spaces that include airports, apartment areas, schools, and even hospitals [1,2]. Additionally, they require time-consuming, labor-intensive blood tests for correctly diagnosing illness symptoms, preventing the spread of viral particles from being effectively stopped.

Paper microfluidic electrochemical devices almost perfectly meet this need, while allowing an external power-free separation of a sample solution, and selective detection of target materials through the microfluidic channel at one time. Due to their low cost, effectiveness, and convenience for field-based point-of-care detection, the paper microfluidic devices are excellent analytical tools, especially in electrical power-deficient underdeveloped countries [3,4,5,6]. Recently, even more advanced versions of the paper devices have been introduced by combination with surface-enhanced Raman spectroscopic substrates, or with enzyme-linked immunosorbent assay [7,8]. In addition, two-dimensional and three-dimensional paper devices have been reported [3]. Glassy carbon and conducting polymer-based multidetection of human blood ions has been introduced [9]. However, direct detection of blood ions is still difficult and simultaneous detection of major human blood ions is even more challenging, because of the many other interfering materials, such as red blood cells, white blood cells, proteins, and hormones. These biological and chemical blockers coexist in the blood stream, and effectively screen precise detection of the ions [10,11].

Non-paper-based simultaneous detection and paper-based non-simultaneous detection of human blood ions have been introduced [9,11]. In this work, we integrate, for the first time, a wax-patterned paper microfluidic substrate, three cation permeable thin films allowing exclusive pathways for sodium, calcium, or potassium ions, and plastic cover plates into a paper-based total analysis system to simultaneously determine major human blood ions. Optimization and characterization of the wax patterns are conducted in terms of potentiometric sensitivity and response time. The major human blood ions, including Cl^−^, K^+^, Na^+^, and Ca^2+^, are physiological indices as indicators of human body disorders. Thus, an accurate determination of blood ions is of great importance to assess whether numerous metabolisms and homeostasis in the human body are working correctly.

## 2. Materials and Methods

### 2.1. Conceptual Theories

Zero current potential caused by concentration difference of chloride ions between the reference and sample solutions logarithmically depends on the chloride ion concentration of the sample solution, and the electrochemical reversible reaction that is involved (Equation (1)) is governed by the corresponding Nernst equation (Equation (2)):

(1) $$\mathrm{AgCl}(s)+e^{-}⇌\mathrm{Ag}\left(s\right)+Cl^{-}$$

(2) $$E=E^{0\prime }-\frac{\mathrm{RT}}{\mathrm{nF}}\ln\left[Cl^{-}\right]$$

where E^0^′, R, T, F, and n represent the formal potential (V), gas constant, temperature (K), faraday constant (C mol^−1^), and the number of electron (mol), respectively. 

Similarly, concentration of potassium ion can be described as a function of zero current potential by the use of potassium permeable film. Chloride ions in this case cannot be supplied through the salt bridge, and the chloride ion concentration in the sample solution side is practically constant. Therefore, aqueous potassium chloride and silver/silver chloride-originated chloride ions should be in equilibrium (Equation (3)). The resulting Nernst equation is also shown below (Equation (4)):

(3) $$K^{+}+Cl^{-}+e^{-}⇌Cl^{-}+K\left(s\right)$$

(4) $$E=E^{0\prime }-\frac{\mathrm{RT}}{\mathrm{nF}}\ln\frac{\left[Cl^{-}\right]}{\left[K^{+}\right]\left[Cl^{-}\right]}.$$

Because the chloride ion concentrations shown in logarithmic term are canceled, the zero current potential E is a function of the potassium ion concentration only. 

Determination of sodium ion concentration is exactly the same as that of potassium ion, except sodium permeable film is used, and the electrolyte is sodium chloride instead of potassium chloride, i.e., aqueous sodium chloride and silver/silver chloride-originated chloride ions should be in equilibrium (Equation (5)). The resulting Nernst equation is as shown in Equation (6):

(5) $$Na^{+}+Cl^{-}+e^{-}⇌Cl^{-}+\mathrm{Na}\left(s\right)$$

(6) $$E=E^{0\prime }-\frac{\mathrm{RT}}{\mathrm{nF}}\ln\frac{\left[Cl^{-}\right]}{\left[Na^{+}\right]\left[Cl^{-}\right]}.$$

In this case, the zero current potential E is a function of the sodium ion concentration only.

Potentiometric measurement of calcium ion concentration is the same as described above, except the calcium permeable film and CaCl_2_ electrolyte should be used. The electrochemical reversible reaction and the Nernst equation are presented here as Equations (7) and (8), respectively:

(7) $$Ca^{2+}+2Cl^{-}+2e^{-}⇌2Cl^{-}+\mathrm{Ca}\left(s\right)$$

(8) $$E=E^{0\prime }-\frac{\mathrm{RT}}{\mathrm{nF}}\ln\frac{{\left[Cl^{-}\right]}^{2}}{\left[Ca^{2+}\right]{\left[Cl^{-}\right]}^{2}}.$$

**Figure 1:**
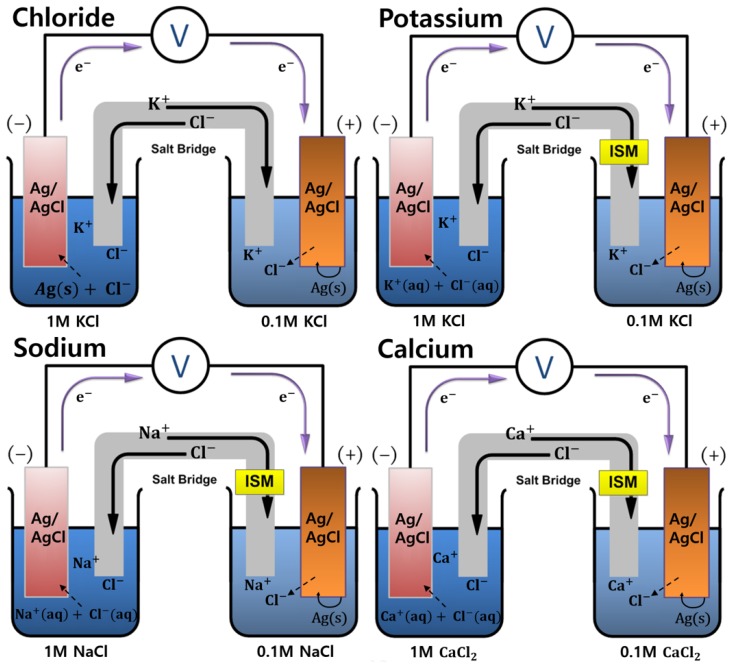
Drawing of electrochemical cell configurations showing theoretical background in the detection of blood ions, i.e., Cl^−^, K^+^, Na^+^, and Ca^2+^. Note that ISM represents ion-selective membranes for each cations.

The electromotive force (EMF) of potassium, sodium, and calcium cations linearly increases as each cation increases in a logarithmic manner, because cations are shown as denominators in the logarithmic terms. Chloride ion is shown as the numerator in the logarithmic term, and the cell potential decreases as the chloride ion concentration increases. Figure 1 shows a schematic of the electrochemical cell configuration for the detection of each blood ion. Note that the formal potential, which is the sum of the standard potential and a logarithmic term of the activity coefficient ratio of the involved redox species, varies from person to person because the ionic strength of blood serum strongly affects the activity coefficients [12].

### 2.2. Fabrication of the Paper Microfluidic Components

Functional films to permeate selective ions commonly contain polyvinyl chloride (PVC, high molecular weight, Sigma-Aldrich, Milwaukee, WI, USA) as a polymer backbone, 2-nitrophenyl octyl ether (NPOE, Sigma-Aldrich, Milwaukee, WI, USA) as a lipophilic plasticizer, and potassium tetrakis(4-chlorophenyl)borate (Sigma-Aldrich, Milwaukee, WI, USA) as an electrolyte to adjust the hydrophilicity of the film. Additionally, potassium ionophore I (Sigma-Aldrich, Milwaukee, WI, USA) for K^+^, 4-tert-butylcalix[4]arenetetraacetic acid tetraethyl ester (Sigma-Aldrich, Milwaukee, WI, USA) for Na^+^, and *N*,*N*,*N*′,*N*′-tetracyclohexyl-3-oxapentan ediamide (Sigma-Aldrich, Milwaukee, WI, USA) for Ca^2+^ are employed as additives, which enable the selective detection of blood ions. Some representative ion-selective electrode materials have been reported [13]. Each solution mixture to prepare the functional film is dried for 24 h under atmospheric condition at room temperature, after adding volatile tetrahydrofuran (Sigma-Aldrich, Milwaukee, WI, USA) for better homogeneity of the products. Table S1 lists the actual compositions of the solution mixtures. 

**Figure 2:**
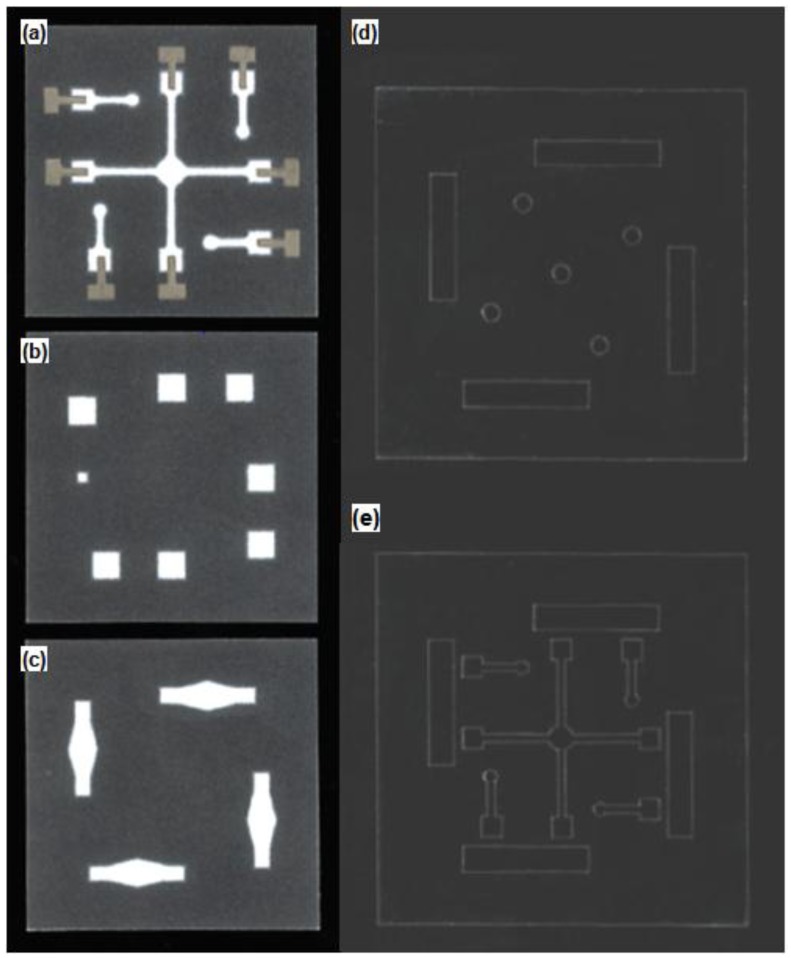
Photo images of the paper-based (**a**) channels (**b**) electrode wells, and (**c**) salt bridges. (**d**,**e**) The first and second cover tops prepared for easy sample loading and faster solution transport, respectively. Note that the plastic bottom plate is omitted for simplicity.

Figure 2a–c show the wax-patterned paper sheets employed in this work. One is to prepare paper channels, another one for reference and sensing electrode wells, and the other one for salt bridges connecting each reference and corresponding sensing electrode pairs, respectively. Note that waxed areas are shown in black. Four reference electrode areas that are supposed to be soaked with different composition of solutions are isolated from each other and from the sensing electrodes area. Sample solution for sensing electrodes may be dropped down right above the center circle and flowing through the assigned paper-based microchannels. To create the paper channels, the solid wax-patterned Whatman filter paper (grade 1, 180 μm, GE Healthcare, Chicago, IL, USA) is treated at 130 °C for 45 s, allowing the melted wax to penetrate deep inside the porous cellulose paper by capillary action. Biocompatible solid wax patterning of the paper is performed with a laser printer (Fuji Xerox, Tokyo, Japan). Each electrode well has the same size (4.5 × 4.5 mm), except the sensing electrode well (2 × 2 mm) for the detection of chloride ion, which does not need any ion permeable membrane. The reduced well size slows down the fluid flowing speed in the chloride ion sensing channel to be similar with the flowing speeds in the other channels. Note that detection of K^+^, Na^+^, and Ca^2+^ ions requires specific ion-permeable membranes, which commonly hinder the free movement of solutions in the channel. The hydrophobic wall-mounted paper electrode substrate by wax printing is combined with one-sided or double-sided adhesive poly(ethylene terephthalate) (PET, Copierland, Seoul, Korea) films. Figure 2d,e show the top and the second top PET cover films. The top layer has five round shaped holes: one for loading a sample solution (~150 μL); the others for loading reference solutions (~50 μL each). Four rectangularly shaped holes are prepared for electrical connection to external circuits. The second layer is engraved with the same patterns of the underneath wax-patterned paper and superimposed on the paper layer. By combining with the top layer, the second layer provides a small volume of empty space right above the paper channel, which enables faster transfer rates of reference and sensing solutions. Note that punching of the PET film is performed with a laser cutter (Silhouette, Orem, UT, USA).

**Figure 3:**
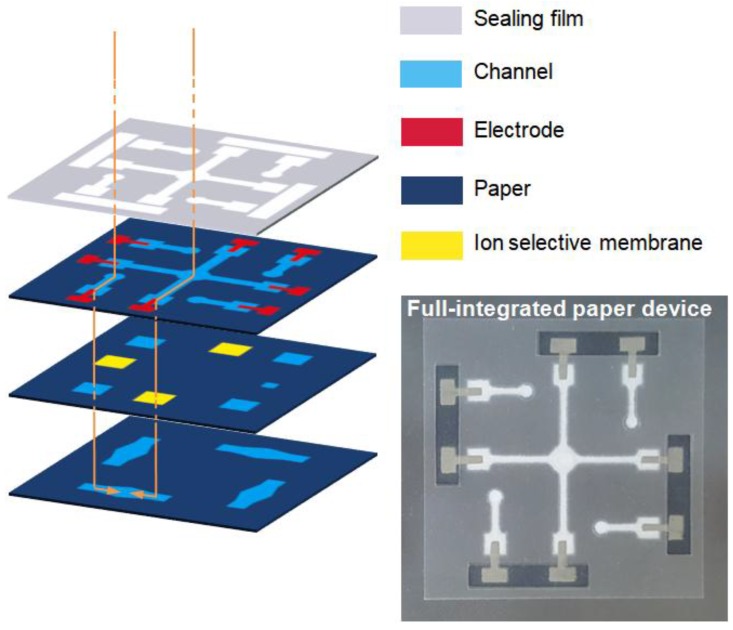
Configuration of paper and plastic layers composing the fully integrated paper-based eletrochemical device. Photo image of the assembled device clearly shows Ag/AgCl electrodes and paper channels through the transparent plastic covers.

Figure 3 shows the configuration of a multi-layered paper microfluidic electrochemical device composed of three paper and three PET layers, and the real photo image. Ion selective membranes are embedded between paper layers. Ag/AgCl electrode probes that should be positioned on each reference and sensing electrode wells are prepared by screen-printing Ag/AgCl ink (Ag:AgCl = 50:50 in weight, Gwent, Pontypool, UK) and subsequently heat-cured at 90 °C for 6 min to dry. Paper channels are then oxygen plasma treated to enhance the surface hydrophilicity. Zero current potentials for each reference and sensing electrode pairs are measured using a digital voltmeter (Keithley, Solon, OH, USA). 

## 3. Results

### 3.1. Structural Optimization of Paper Microfluidic Systems

Three different inlet shapes for sample loading with basic geometries, such as a circle (diameter = D), a regular square (side length = D), and a triangle (side length = D), are considered (Figure S1). The hydraulic radius (r_H_) defined as r_H_ = A/P (where A is the fluid channel’s cross-sectional area, and P is the wetted perimeter of the channel) indicates that the hydraulic diameter of the regular triangle is about 58%, as compared with those of the circular or the regular square, because the hydraulic diameter is defined as four times the r_H_. This means that the pressure force of a sample solution loaded through the triangle inlet acting on unit surface area is larger than the others, assuming a constant sample volume. Therefore, it is possible to obtain a higher fluid flow rate with the triangle inlet and a higher Reynolds number (Re), which is defined as Re = (D_H_ρv)/μ, where v is the fluid velocity (m s^−1^), and ρ and μ are the density (kg m^−3^) and viscosity (kg m^−1^ s^−1^) of the fluid, respectively. However, a turbulent flow in a microchannel is unobtainable, because typically the flow within such a small channel restricts Reynolds numbers to between 10^−5^ and 10^−3^, so a laminar flow may govern the entire fluid flow [14]. Additionally, boundary layers between fluid streamlines are not available due to the porous paper channel, and a plug flow will be dominantly observed. In conclusion, the sample inlet shape does not appreciably affect the fluid flow mechanism in the paper-based microchannel as shown in Figure S1. Circular inlets were chosen for convenient loading of the sample solution. 

### 3.2. Shape of the Salt-Bridged Mixing Channel

The theoretical function of a salt bridge is to electrically connect the two electrolytes having different compositions or concentrations with each other, by the movement in or out of ions from the salt bridge. Because the salt bridge acts as an ion source for neutralizing each half cell electrolyte, it should only contain highly concentrated ions. In this work, the salt bridge area acts not only as the electrical connector but also as an actual mixing area for loaded solutions coming from both solution inlets. This means that a thick diffusion layer will be formed in the mixing channel, and the steeper concentration gradient commonly observed with a wider mixing area will show higher zero current potential. The potential difference between the Ag/AgCl electrodes would decrease and eventually disappear, provided that each layer component of the paper microfluidic device was airtight, and the ions inside were freely moved to reach an electrochemical equilibrium state. Figure S2a,b show that the device with a diamond-shaped mixing channel demonstrates about 1.5% higher maximum zero current potential than that with a rectangular one. 

### 3.3. Heating and Drying Processes for Wax Diffusion

Patterned solid wax on a paper substrate is heated to diffuse deeply into the paper and to form hydrophobic walls to prepare micro channels. The heating and drying processes are optimized by comparing the six different heating and drying processes: two different heating temperatures (90 and 130 °C) and three different drying times (6, 12, and 30 min), producing six different conditions (Figure S3). 

While the drying time at 90 °C does not seriously affect the diffusion length of the wax, the length increases as the drying time increases at 130 °C. An increased diffusion length means that the boundary between the hydrophobic wax and hydrophilic paper is unclear. According to the Washburn equation (L^2^ = (γDt)/(4ν), where L, γ, D, t, and ν represent the penetration length, surface tension, mean pore diameter, penetration time, and dynamic viscosity, respectively), L = 0.83 mm would be returned if human blood serum were flowed through a porous paper medium at 130 °C for 6 min [15]. However, a much longer diffusion length of 1.09 mm can be observed in a circular diffusion because of the concentration gradient that is steeper than that in a planar diffusion. The diffusion area in a circular diffusion decreases as the distance from the curved surface increases. 

Excessive diffusion, either by over heating or by allowing a long duration, can affect the zero current potential output from the device. Under a condition where 0.1 M K^+^ ions are loaded on a circular inlet and flow through the device channel, the potential outputs of Sample Numbers 1, 2, and 3 are reproducible. Sample Number 4 shows unstable and fluctuating signals. Sample Numbers 5 and 6 do not show any signal outputs, indicating that the melted solid wax has penetrated deeply into the micro channels and has electrically disconnected them. 

### 3.4. Oxygen Plasma-Based Surface Hydrophilization

The micro channels built of hydrophobic wax require post-treatment of the surface, to be hydrophilic for faster fluid flow of an aqueous medium. Basically, the surface modification processes are categorized into two major groups, which are oxygen plasma-based physical treatments and chemical treatments by the use of bifunctional chemicals, such as 3-aminopropyl dimethyl ethoxysilane (APDMES) [16]. Even though the physically prepared hydrophilic surface shows a relatively short lifetime, the oxygen plasma treatment is widely used, because the plasma-induced chemical functional groups on the target surface are delocalized over the entire surface [17,18]. However, the use of oxygen plasma to modify hydrophobic wax walls in a paper cellulose-packed environment is ineffective. Instead, APDMES treatment of the paper device by dipping is utilized in this work, and results are obtained that are comparable with the plasma treatment. Figure S4 shows different signal output patterns of non-treated, oxygen plasma (O_2_ Plasma Cleaner, Femto Science Laboratory, Seoul, Korea)-treated, and APDMES (Sigma-Aldrich, Milwaukee, WI, USA)-treated PAD channels. The surface modified channels show higher zero current potentials and faster responses than the non-treated channel. 

### 3.5. Performance Characterization of Paper-Microfluidic Devices by Electrochemical Potentiometry

In electrochemical potentiometry, the actual current flow between the reference and sensing electrodes is almost zero, and only a negligible amount of current flow is allowed to measure the potential difference between two different electrolyte solutions with different ionic strength. In this work, the zero current voltage output is recorded with an oscilloscope for 10 min, and the maximum voltage during that period of time is selected to be representative of the concentration of a target ion. Commonly, response times to all target ions are within 30 s. 

**Figure 4:**
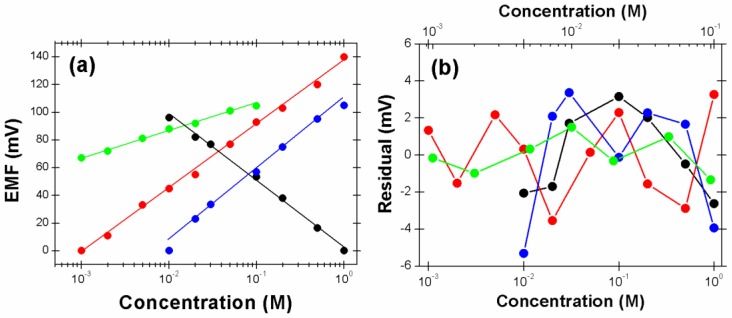
(**a**) Calibration of paper microfluidic devices in standard solutions containing each blood ion. Note that five different paper devices were used to obtain a point in calibration curves (one device can be used only one time), and the error bars were omitted for simplicity. Note that the theoretical slope for single charged ions (Cl^−^ (black), K^+^ (red), and Na^+^ (blue)) is 59.2 mV decade^−1^, while that for doubly charged ion (Ca^2+^ (green)) is 29.6 mV decade^−1^. (**b**) Residual diagrams for calibration curves. Top axis is for red and green curves.

Figure 4a shows calibration curves of the paper microfluidic device to chloride, potassium, sodium, and calcium ions, where the zero current potentials are recorded as a function of ion concentrations. While the reference solution for Cl^−^ detection is 1 M KCl, the concentration of KCl in the sensing solution varies from 0.01 to 1 M. The sensitivity of chloride anion is −47.71 mV decade^−1^ (R^2^ = 0.9968), 21.6% less than the theoretical 59.2 mV decade^−1^ that is probably due to the distorted electrochemical behavior of the screen-printed Ag/AgCl electrode. The Ag/AgCl electrode is prepared by heat curing of the screen-printed silver ink and low purity of the screen-printed silver causes the sensitivities to be lower than that expected by the Nernst equation. The deviation becomes smaller as the ion concentrations near the two Ag/AgCl electrodes come closer [12]. The available detection range of chloride anion is relatively small as compared with previous data [19]. However, the calibration curve shows a short response time and a linear response in the range. For the calibration of potassium, 10^−3^ M KCl is used as the reference solution, and the linear concentration range of KCl in the sensing solution is between 10^−3^ and 1 M. The measured potentiometric sensitivity to K^+^ is 45.97 mV decade^−1^ (R^2^ = 0.9973), 22.3% less than the theoretical value. In the case of sodium, the reference solution is 1 M NaCl, and the linear concentration range of NaCl in the sensing solution is between 10^−3^ and 1 M. The measured response to sodium ion is 51.06 mV decade^−1^ (R^2^ = 0.9912), 13.7% less than the theoretical value. The sensitivity of the device to calcium cation is 19.46 mV decade^−1^ (R^2^ = 0.9916) in a linear range between 10^−3^ and 10^−1^ M of CaCl_2_, which is 34.1% smaller than the theoretical value. Note that the calcium ion is a doubly charged cation, and the theoretical zero current potential increases 29.6 mV per decade concentration increase of Ca^2+^. The paper device presented in this work shows a wide linear detection range available for the direct analysis of physiologic human blood ions, because the normal concentration ranges of Cl^−^, K^+^, Na^+^, and Ca^2+^ in human serum samples are 98~109 mM, 3.5~4.9 mM, 138~146 mM, and 1.12~1.32 mM, respectively [20]. The small fitting deviation (Figure 4b) indicates that the device presented in this work is carefully optimized and reproducible to analyze blood ions.

**Figure 5:**
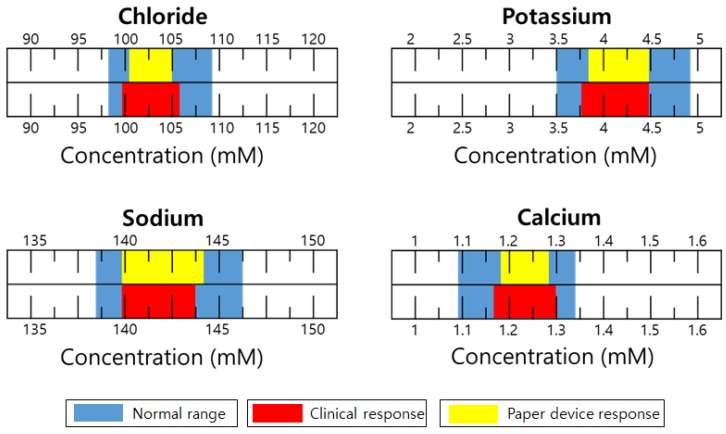
Comparison of potentiometric responses to target ions of the paper microfluidic device and the Abbott i-Stat. Note that clinical data were supported by the Seegene medical foundation, and only normal serum samples from human blood were available outside the hospital.

Figure 5 shows a practical application of the paper device to unknown serum samples. Normal serum samples from human blood were supported by the Seegene Medical Foundation (Seoul, Korea). Each blood ion concentration was determined 10 times by 10 different devices, and with the exception of the maximum and minimum numbers, and the resulting eight numbers were averaged. The reference solutions for Cl^−^, K^+^, Na^+^, and Ca^2+^ were 1 M KCl, 10^−3^ M KCl, 10^−1^ M NaCl, and 10^−2^ M CaCl_2_, respectively. The results show that the output data from the paper devices are in the normal ranges of each cation and even comparable to the commercially available clinical data that are obtained from i-Stat (Abbott, Lake Bluff, IL, USA). However, the potentiometric signal output of the device for human serum ions is not exactly the same as that determined by commercial clinical equipment. This means that a correlation curve is needed for connecting the two measurement systems. The discrepancy shown in these two systems is basically due to insufficient filtration of blood samples prior to use of the paper device. Real blood requires complicated routine filtration or prefiltration steps to eliminate numerous biomacromolecules and blood cells to minimize interfering effects.

## 4. Discussion

In this work, we prepared a fully integrated electrochemical paper-based total analysis system using the cellulose fiber of a paper substrate as a natural nano filtering structure for blood serum to minimize the interference effect for a more accurate analysis of human blood ions in the field. Furthermore, integration of all four pairs of reference and sensing electrodes into an analysis system enables a small sample volume and reduced time and effort. The ionic concentration polarization that was induced between the two sides of the salt bridged mixing area (in the case of Cl^−^ detection), or the cation permeable film-integrated mixing component (in the cases of K^+^, Na^+^, and Ca^2+^ detection), causes electrochemical potential, and the potential difference between the reference and sensing electrodes can be potentiometrically quantified via the Nernst equation that governs an electrochemical equilibrium state. 

The wax-patterned paper platform presented herein is for a cost-effective field test device with wide applicability to any blood ion, provided that the platform is correctly combined with the required cation permeable films. The preparation of a hydrophobic channel barrier and hydrophilic treatment of the inner surface of the barrier with oxygen plasma enable volume control of a serum sample. The simple fabrication and the lack of need for an external power source for the paper device are compatible with ubiquitous point-of-care systems and availability for low budget mass production. Excellent reproducibility, the relatively precise determination of blood ion concentration via correlation curves, and the short response time of electrochemical potentiometric measurement indicate that the paper microfluidic electrochemical device has a promising future as a high-accuracy single-use biosensor for end users at any time and at any location, even though a fully working mechanism and variables including selectivity coefficients and the permeability of the membrane components in the paper device are not yet completely understood. We also believe that, because no biodegradable materials are employed for the device, the paper device will be available for many years if the paper device presented herein is offered in a lidded plastic bottle containing a water-content-controlling agent. Further works may include the preparation of a wider lineup of low-cost cation permeable films for analyzing numerous blood ions, and the correlation curve-free calibration for faster operation, which would allow for greater sensitivity and selectivity of human blood ions. 

## Supplementary Materials

The following are available online at http://www.mdpi.com/1424-8220/18/1/104/s1. Figure S1: Photo images showing the different fluid loading patterns but the similar flow rates (channel length = 10 mm) (A), and the coffee effect in the patterns (B). (C) A plot of fluid flow distance as a function of time. Note that a universal indicator solution for pH measurement is used to clearly see the solution flow with naked eye, Figure S2: Dependence of EMF on the shape of the electrolyte mixing channel at various concentrations (10^−3^~10^0^ M KCl) of the sample solutions. Note that injection volumes of reference and sample solutions are 5 μL, and the reference solution is 10^−3^ M KCl, Table S1: Summary of reagents used for preparing each ion selective membrane.
