# Supplementary material for: A Fully Integrated Paper-Microfluidic Electrochemical Device for Simultaneous Analysis of Physiologic Blood Ions
Source: Sensors (Basel). 2018 Jan 1;18(1):104. doi: 10.3390/s18010104 (PMC5796313; doi:10.3390/s18010104)
Supplement: Supplementary file 1 [file sensors-18-00104-s001.pdf]

Supplementary Material

A Fully Integrated Paper-Microfluidic Electrochemical Device for Simultaneous Analysis of Physiologic Blood Ions

Joon-Hyung Jin [1], Joon Hyub Kim [2], Sang Ki Lee [2], Sam Jin Choi [3], Chan Won Park [4,*] and Nam Ki Min [2,*]

[1] Department of Chemical Engineering, Kyonggi University, 154-42 Gwanggyosan-ro, Yeongtong-gu, Suwon-si, Gyeonggi-do 16227, Korea; jjh1023@kgu.ac.kr

[2] Department of Control and Instrumentation Engineering, Korea University, 2511 Sejong-ro, Sejong City 339-770, Korea; kim4539@korea.ac.kr (J.H.K.); snagki0@korea.ac.kr (S.K.L.)

[3] Department of Biomedical Engineering, College of Medicine, Kyung Hee University, Seoul 130-701, Korea; medchoi@khu.ac.kr

[4] Department of Electrical and Electronic Engineering, Kangwon National University, Chuncheon 200-701, Korea

* Correspondence: cwpark@kangwon.ac.kr (C.W.P.); nkmin@korea.ac.kr (N.K.M.)

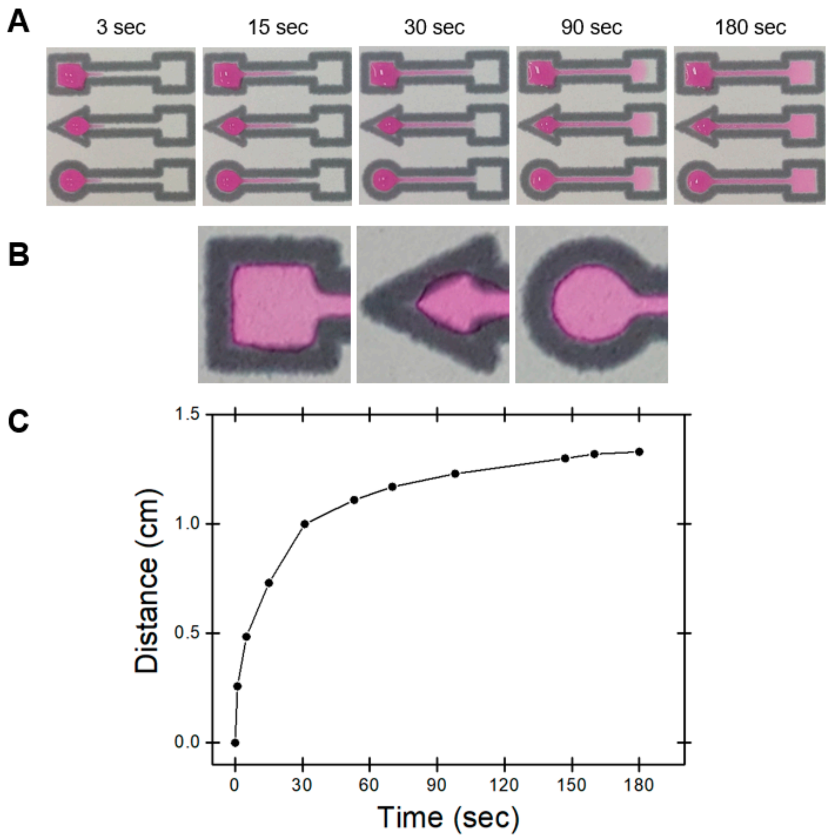

Figure S1. Photo images showing the different fluid loading patterns but the similar flow rates (channel length = 10 mm) (A), and the coffee effect in the patterns (B). (C) A plot of fluid flow distance as a function of time. Note that a universal indicator solution for pH measurement is used to clearly see the solution flow with naked eye.

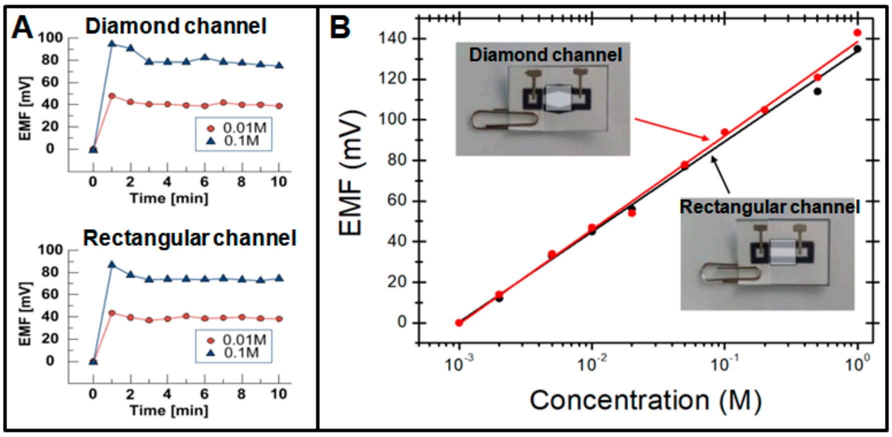

Figure S2. Dependence of EMF on the shape of the electrolyte mixing channel at various concentrations (10^{-3}~10^{0} M KCl) of the sample solutions. Note that injection volumes of reference and sample solutions are 5 μL, and the reference solution is 10^{-3} M KCl.

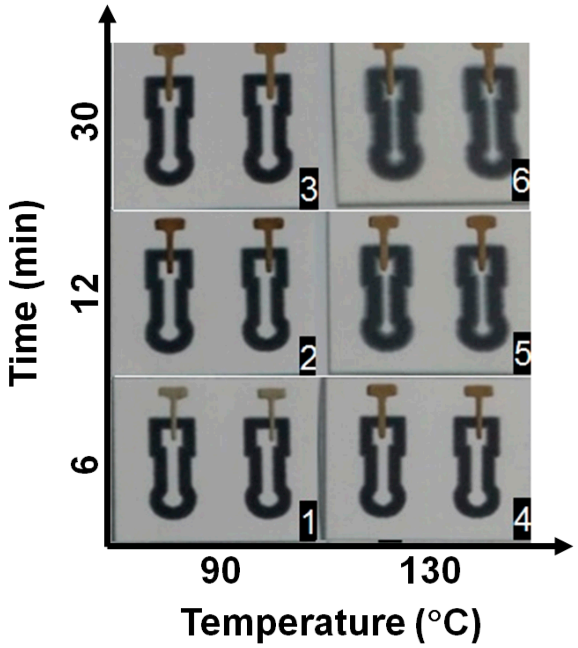

Figure S3. Photo images of sample and reference solution channels prepared at conditions 1 (90 °C, 6 min), 2 (90 °C, 12 min), 3 (90 °C, 30 min), 4 (130 °C, 6 min), 5 (130 °C, 12 min), and 6 (130 °C, 30 min). Note that line pattern shown in this figure is designed exclusively for optimization of heating and drying processes for wax diffusion.

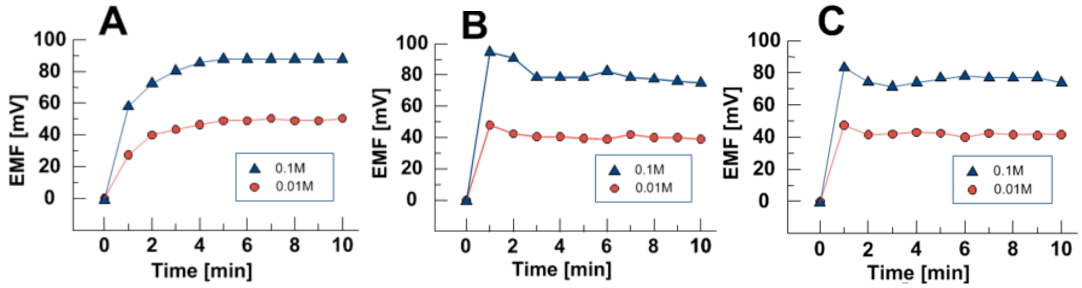

Figure S4. EMF characteristics of (**a**) non-treated, (**b**) oxygen plasma-treated, and (**c**) APDMES-treated micro channels. Note that 10^{-1} M or 10^{-2} M KCl solutions are loaded through a circular inlet.

Table S1. Summary of reagents used for preparing each ion selective membrane.

Target ion	Reagents	Weight percent	Weight or volume
K^+	Potassium ionophore I	1.5 wt%	14 mg
	Potassium tetrakis(4-chlorophenyl)borate	0.5 wt%	3 mg
	Polyvinyl chloride (PVC, high molecular weight)	33 wt%	328 mg
	2-Nitrophenyl octyl ether (NPOE)	65 wt%	0.63 mL
Na^+	4-tert-Butylcalix[4]arenetetraacetic acid tetraethyl ester	1 wt%	9.9 mg
	Potassium tetrakis(4-chlorophenyl)borate	0.5 wt%	2.5 mg
	Polyvinyl chloride (PVC, high molecular weight)	33 wt%	329 mg
	2-Nitrophenyl octyl ether (NPOE)	65.5 wt%	0.63 mL
Ca^{2+}	N,N,N′,N′-tetracyclohexyl-3-oxapentanediamide	1 wt%	10 mg
	Potassium tetrakis(4-chlorophenyl)borate	0.5 wt%	6 mg
	Polyvinyl chloride (PVC, high molecular weight)	33 wt%	328 mg
	2-Nitrophenyl octyl ether (NPOE)	65.5 wt%	0.63 mL
